# Annotations

**Published:** 1910-06-18

**Authors:** 


### ANNOTATIONS.

### Sir William Macewen.

Certain proceedings are attributed to Sir William Macewen at the time of King Edward's death, which are stated to have been as follows:—The authorities of the Western Infirmary, Glasgow, following the precedent for nurses and other members of the staff of hospitals throughout the country, which was set when Queen Victoria died, supplied black bands to be worn by the nurses on the left arm. All the staff wore these bands, and the patients in the wards, we are informed, were much touched by this mark of respect for their dead King. In Sir William Macewen's wards, however, by his orders, the nurses had to remove these mourning bands, and the wards of this surgeon to the late King in Scotland were the only section of this important hospital which did not contain these signs of mourning for King Edward VII. The position of the nurses in Sir William Macewen's wards must have been painful. Indeed, the nurses, it is stated, carried the mourning bands in their pockets, and directly they were outside Sir William's wards they replaced them, and wore them throughout the rest of each day, for they yielded to no one in their feelings of loyalty to the throne. We refrained from publishing these particulars when they reached us before the funeral, for they could not fail to prove painful reading for every loyal man and woman, and King Edward was always so full of consideration and thoughtfulness for others. The appearance of Sir William Macewen's name on the list of medical appointments to the household of King George the Fifth has brought once more into prominence the action attributed to Sir William in forbidding his nurses to wear the official mourning for our late beloved King. We feel, therefore, it is due to medical and public opinion that we should publicly ask Sir William Macewen if the facts given above are correct?

### Medicine and the Royal Academy.

The medical profession is well illustrated among the portraits on the walls of this year's Academy. The two Royal Colleges are represented by Sir Henry Morris and Dr. Edward Liveing, and Mr. Ouless's picture of the former is perhaps the best male portrait in the collection this year. Medicine and politics find exposition in Mr. Henry Pegram's bronze bust of Dr. Alfred Hillier, M.P., which has been placed in the Lecture Room. The Treasurer of Guy's Hospital is so closely allied by his position to medicine that we cannot pass on without noticing Mr. Herbert Draper's excellent portrait of this well-known hospital worker. The artist has happily caught that expression of genial good nature associated with firmness and fine business acuity which all who know Mr. Cosmo Bonsor recognise as being so characteristic of the man. Medical men will find much to interest them in this year's collection. apart from any artistic value that the pictures possess. There is little or nothing to cavil at in the anatomical proportions of the various figures. The lower limbs, perhaps, are disproportionately large to the rest of the figure in Mr. Dampier May's picture, "The Dew" (486), while a curve backwards where the occiput joins the neck is needed in Mr. Frank Gatter's statuette of Cain (1738). But on the whole, glaring anatomical mistakes are conspicuously absent. The story of Adam and Eve seems to be one of great interest to sculptors, for there is this year a group representing Eve tempting Adam, and no fewer than three frescoes of the expulsion from the Garden of Eden. Yet all the artists miss the physiological meaning of the two sentences: "I will greatly multiply thy sorrow and thy conception: in sorrow shalt thou bring forth children," and "Adam called his wife's name Eve, because she was the mother of all living." These two together seem to suggest that at the time of the expulsion Eve was advanced in her pregnancy and expecting to be confined. Yet we only know one artist who has recognised this. In a Limoges enamel of the sixteenth century at the Wallace collection Eve is represented as being six months pregnant (Gallery III., No. 250). We do not know whether Botticelli ever painted this subject; if so he probably followed his rule and made the female figure a pregnant one. We regret to see the cult of the fig-leaf still in vogue. Either the representation of the nude is immoral or it is not, and we cannot understand the consistency of the man who fashions a nude figure and then covers up the external genitalia by gumming a fig-leaf on them. We are glad to say that Mr. Hamo Thornycroft has not done this in his beautiful statuette of "A Bather" (1860), though Mr. Charles L. Hartwell has spoilt one of the best representations of athletic youth that we have ever seen at the Academy in this way. In this latter case—"The Victor" (1904)—the absurdity is enhanced by it being perfectly apparent that the particular leaf employed would be inadequate for the purpose in a man of such physique. To cover the genitalia with a fig-leaf is far less likely to pacify the objections of a prude than to draw attention to what the covering is meant to hide and to raise up suggestions in minds that would otherwise never entertain them. Two of our artists have recognised this, and have imitated the fashion of the gauze veils, so often worn by nymphs, by making the leaf a part of a vine tendril which happens to be straggling in that direction (1680, 1703). We sincerely trust that in next year's exhibition this last trace of puritanical influence will be removed from British art.

